# Fighting Cancer around the World: A Framework for Action

**DOI:** 10.3390/healthcare10112125

**Published:** 2022-10-25

**Authors:** Denis Horgan, Rizwana Mia, Tosan Erhabor, Yosr Hamdi, Collet Dandara, Jonathan A. Lal, Joel Fokom Domgue, Oladimeji Ewumi, Teresia Nyawira, Salomé Meyer, Dominique Kondji, Ngiambudulu M. Francisco, Sadakatsu Ikeda, Chai Chuah, Roselle De Guzman, Anupriya Paul, Krishna Reddy Nallamalla, Woong-Yang Park, Vijay Tripathi, Ravikant Tripathi, Amber Johns, Mohan P. Singh, Maude E. Phipps, France Dube, Kate Whittaker, Deborah Mukherji, Hadi Mohamad Abu Rasheed, Marta Kozaric, Joseph A. Pinto, Stephen Doral Stefani, Federico Augustovski, Maria Eugenia Aponte Rueda, Ricardo Fujita Alarcon, Hugo A. Barrera-Saldana

**Affiliations:** 1 European Alliance for Personalised Medicine, 1040 Brussels, Belgium; marta.kozaric@euapm.eu; 2 Department of Molecular and Cellular Engineering, Jacob Institute of Biotechnology and Bioengineering, Sam Higginbottom University of Agriculture, Technology and Sciences, Prayagraj 211007, India; jonathanalal@shiats.edu.in (J.A.L.); vijay.tripathi@shiats.edu.in (V.T.); 3 Grants, Innovation & Product Development, South African Medical Research Council, Francie Van Zijl Drive, Parow Valley, Cape Town 7505, South Africa; rizwana.mia@mrc.ac.za; 4 Medical Laboratory Science Council of Nigeria (MLSCN), Durumi, Abuja 900110, Nigeria; tosanmelody@yahoo.com; 5 Laboratory of Biomedical Genomics and Oncogenetics, Institut Pasteur de Tunis, University of Tunis El Manar, Tunis 1002, Tunisia; yosr.hamdi@pasteur.tn; 6 Laboratory of Human and Experimental Pathology, Institut Pasteur de Tunis, Tunis 1002, Tunisia; 7 Division of Human Genetics, Department of Pathology, Institute of Infectious Disease and Molecular Medicine (IDM), University of Cape Town, Observatory, Cape Town 7925, South Africa; collet.dandara@uct.ac.za; 8 Institute for Public Health Genomics, Department of Genetics and Cell Biology, GROW School of Oncology and Developmental Biology, Faculty of Health, Medicine and Life Sciences, Maastricht University, 6211 LK Maastricht, The Netherlands; 9 Departments of Epidemiology, and Gynecologic Oncology and Reproductive Medicine, The University of Texas MD Anderson Cancer Centre, Houston, TX 77030, USA; jfokom@mdanderson.org; 10 Department of Obstetrics and Gynecology, Faculty of Medicine and Biomedical Sciences, University of Yaounde, Yaounde VF7W+4M9, Cameroon; 11 Freelance Health Care, Life Sciences, Medical Artificial Intelligence Content Writer, Lagos 100253, Nigeria; ola@thehealthandaiwriter.com; 12 National Commission for Science, Technology and Innovation in Kenya (NACOSTI), Nairobi 00100, Kenya; teresia.nyawira@nacosti.go.ke; 13 Cancer Alliance, Cape Town 7700, South Africa; salomefaan@gmail.com; 14 Health & Development Communication, Building Capacities for Better Health in Africa, Yaounde P.O. Box 2032, Cameroon; dkondji@gmail.com; 15 Grupo de Investigação Microbiana e Imunológica, Instituto Nacional de Investigação em Saúde (National Institute for Health Research), Luanda 3635, Angola; franciscongiamb@yahoo.com; 16 Department of Precision Cancer Medicine, Tokyo Medical and Dental University, Tokyo 113-8510, Japan; ikeda.canc@tmd.ac.jp; 17 Singularity University, P.O. Box 165, Gold Coast, QLD 4227, Australia; chai.chuah@gmail.com; 18 Oncology and Pain Management Section, Manila Central University–Filemon D. Tanchoco Medical Foundation Hospital, Caloocan 1400, Philippines; deguzmanmd@hotmail.com; 19 Department of Mathematics and Statistics, Faculty of Science, Sam Higginbottom University of Agriculture, Technology and Sciences, Prayagraj 211007, India; anupriya.paul@shiats.edu.in; 20 ACCESS Health India, Hyderabad 500086, India; krishna.reddy@accessh.org; 21 Samsung Genome Institute, Samsung Medical Centre, Sungkyunkwan University, Seoul 06351, Korea; woongyang@skku.edu; 22 Ministry of Labor, Health Department Government of India, New Delhi 110001, India; rkt.mmc@gmail.com; 23 Garvan Institute of Medical Research and the Kinghorn Cancer Centre, Cancer Division, Sydney, NSW 2010, Australia; amber@icgc-argo.org; 24 Centre of Biotechnology, University of Allahabad, Allahabad 211002, India; mpsingh16@allduniv.ac.in; 25 Jeffrey Cheah School of Medicine and Health Sciences, Monash University Malaysia, Subang Jaya 47500, Selangor, Malaysia; maude.phipps@monash.edu; 26 Astra Zeneca, 1800 Concord Pike, Wilmington, DE 19803, USA; france.dube@astrazeneca.com; 27 Cancer Council Australia, Sydney, NSW 2000, Australia; kate.whittaker@cancer.org.au; 28 Global Health Institute, American University of Beirut, Beirut VFXP+7QF, Lebanon; dm25@aub.edu.lb; 29 Department of Hematology/Oncology, American University of Beirut Medical Centre, Beirut P.O. Box 11-0236, Lebanon; 30 Qatar Cancer Society, Doha 22944, Qatar; hadi@qcs.qa; 31 Centre for Basic and Translational Research, Auna Ideas, Lima 15036, Peru; jpinto@gecoperu.org; 32 UNIMED RS, Porto Alegre 90040-180, Brazil; stephens@terra.com.br; 33 Health Technology Assessment and Health Economics, Department of the Institute for Clinical Effectiveness and Health Policy (IECS-CONICET), Buenos Aires C1056ABH, Argentina; faugustovski@iecs.org.ar; 34 Venezuelan Breast Cancer Research and Education Foundation, Caracas 1060, Venezuela; maruaponte@gmail.com; 35 Centro de Genética y Biología Molecular, Universidad de San Martín de Porres, Lima 15024, Peru; rfujitaa@usmp.pe; 36 Innbiogem SC/Vitagenesis SA at National Laboratory for Services of Research, Development, and Innovation for the Pharma and Biotech Industries (LANSEIDI) of CONACyT Vitaxentrum Group, Monterrey 64630, Mexico; habarrera@gmail.com; 37 Schools of Medicine and Biology, Autonomous University of Nuevo Leon, Monterrey 66451, Mexico

**Keywords:** cancer, global, diagnostics, personalized medicine, reimbursement, patients, health care, policy framework, next-generation sequencing, liquid biopsy, genomics

## Abstract

Tackling cancer is a major challenge right on the global level. Europe is only the tip of an iceberg of cancer around the world. Prosperous developed countries share the same problems besetting Europe–and the countries and regions with fewer resources and less propitious conditions are in many cases struggling often heroically against a growing tide of disease. This paper offers a view on these geographically wider, but essentially similar, challenges, and on the prospects for and barriers to better results in this ceaseless battle. A series of panels have been organized by the European Alliance for Personalised Medicine (EAPM) to identify different aspects of cancer care around the globe. There is significant diversity in key issues such as NGS, RWE, molecular diagnostics, and reimbursement in different regions. In all, it leads to disparities in access and diagnostics, patients’ engagement, and efforts for a better understanding of cancer.

## 1. Introduction

According to World Cancer Report 2020, cancer is the first or second leading cause of death before the age of 70 years in 134 of 183 countries and ranks third or fourth in a further 45 countries, with Africa and Asia the regions with the highest premature fatality toll [1]. Europe is currently making efforts to improve its performance in preventing, diagnosing, treating, and caring for the millions of victims attacked by this Medusa of disease, and is beginning to exploit and further develop the tools that can cut off some of the heads of these multiple menaces to lives and life. But this is uphill work even in a prosperous part of the world.

The problems of tackling cancer are, like the disease itself, many-headed [2,3,4]. Increased scientific understanding reveals ever more profound complexities in the manifestations of cancer and the mechanisms behind it. Rapidly advancing technology offers an ever-increasing range of new and potential tools to diagnose and treat in a more personalized manner, wherever the battle is joined [5,6]. Rising attention to and provision of public health care is leading to expanded and more sophisticated efforts for screening at-risk populations and caring for the growing numbers of survivors and the difficulties many of them face during continued monitoring and even after successful treatment [7]. In parallel to the strictly health-related issues, the costs of cancer—in terms of direct costs in diagnosis and treatment, and indirect costs in working lives lost or reduced by disease and by care—present a major challenge in their own right, to the individuals and families affected, but also to the collective and public health systems [8]. Similarly, the organization of care systems, the investment in infrastructure to enable advanced care, the continuation of support for cancer research (including into why some populations are more susceptible than others to particular cancers), and the conduct of large-scale targeted screening are the consequence as much of policy debates and political decisions as the response to scientific or health issues. Still more widely, as the link between cancer and environmental conditions of populations becomes more clear, even broader social considerations relating to matters as diverse as air pollution or housing come into play. 

The multiple components of the challenge demand a multifaceted analysis and response, and since it is policy and politics that ultimately determine most of how society in any country chooses to act and react, the context for this paper has to be the interaction of these many health issues with this harsh reality of top-level decision-making. Whatever the precise characteristics of any country or region, the development and implementation of strategies, including the crucial allocation of resources, will augment, sustain or limit the response to cancer. This is why exponential advances in science, technology, and knowledge in health care are often frustrated by a legacy linear institutional process prone to inconsistency and incoherence, moving at a glacial pace to set the scene for a complex collision between public expectations and delivery of the more precise and personalized health services now becoming increasingly possible. For each health care system, as with each person’s health, the need is to personalize the response to obtain maximum effect [3,9,10]. A one-size-fits-all approach cannot make a sufficient contribution in the age of modern medicine. This is especially true for oncology, where the benefits in improving care are increasingly dependent on the implementation of personalized medicine [11]. Apart from the situation that prevails in each country, it is also crucial to ask questions about the ambitions of the authorities responsible for health services, who should provide them, who will be entitled to which services and who will pay. And beyond these trials are the questions that arise from the background about how stakeholders in the health sector can best support the decision-making process and contribute to improving cancer care [12].

The characteristics of each country and region also inescapably influence the immediate fate or future of cancer patients and the general population [13]. At the acute end, the availability of advanced care with the most modern techniques of genomic medicine and access to effective new therapies–medicines, surgery, and radiology–largely determine outcomes for individuals. Upstream, the care itself is heavily dependent on the availability and quality of the supportive infrastructure, ranging from diagnostic and testing centers and the necessarily associated laboratories, to treatment centers with links to data and tissue banks–and even on consistent electricity supplies to power them, as essential to permit the reality of practicing advanced genetic testing. PM in oncology is not widely practiced even where it is a focus in large cancer centers and those who fund research, because of the cost associated with genomic sequencing and the use of companion diagnostic devices, along with the absence of advanced genetic testing facilities [14,15]. 

Equally essential and intimately related are the specialized professional skills to deliver care, including the multidisciplinary interplay such as in tumor boards. Further upstream still is the way a country organizes its health care system, including the crucial elements of cancer awareness and education of public and professionals, prevention programs, targeted screening strategies, take-up of innovation, access and eligibility, reimbursement, and investment in research, particularly related to local populations. Knowledge of genetics on human health continues to be based predominantly on studies in populations of European ancestry, offering poorer representation of other ethnic populations, including in the developing world. This underrepresentation of many ethnic populations may miss gene–disease relationships for which the exposure or outcome is rare in European populations. Generalizability and the translation of these findings in clinical care are limited [16,17,18].

Ultimately, performance on many of these factors is in turn strongly conditioned by policy decisions and allocation of resources at the highest political level, which brings the circle to completion. The European Beating Cancer Plan (EBCP) and its companion piece, the EU Cancer Mission, are not designed to serve as a model beyond Europe, but they are not without interest for this discussion, in their multi-pronged design, which extends widely either side of treatment into areas of prevention, knowledge, screening, early detection, establishing standards of care, widening access, survivorship, and the particular challenge of childhood cancers [19,20,21]. 

Use of precision oncology together with genomic profiling dramatically improves both diagnosis and targeting therapy in different tumors [22]. However, at a global level, regulatory approval processes and access to testing and to matched drugs vary widely across regions and countries. Analysis of differing uptake of next-generation sequencing (NGS), real-world evidence (RWE), molecular diagnostics and reimbursement systems offers some insights into progress and challenges around the world. Nor is this just an issue of access to new medicines: proper access to health care in all global regions needs respect for fundamental principles aimed at ensuring the health system is appropriate to the social, political, epidemiological, and economic environment [23]. This will also help avoid a possible skewing of development resulting from concentration on only the patients with most resources or access. Integration of expertise in multidisciplinary teams and with molecular tumor boards will help integrate molecular alterations within a clinical context—although logistical challenges can be a barrier [22,24,25,26]. It is a complex moving picture with evolving methods and standards, driving change in the IVD as well as in therapeutics, and requiring greater generation of reliable RWE [27]. 

The aim of this paper was to identify how different global regions are tackling cancer, what the differences are in cancer care around the globe and what mechanisms are used to fight cancer. To contribute to understanding the inevitable complexities, this paper examines, from a range of perspectives, the provision of care and policies relating to cancer, principally across Africa, Asia and the Middle East, and Latin America, with some regional generalizations and some country-specific details. To provide new insights on this topic, the European Alliance for Personalised Medicine (EAPM) organized a series of expert panels where different stakeholders involved in cancer care presented current situations in their countries. The main research question is how to provide a framework for action in battling cancer around the globe.

## 2. Materials and Methods

**Figure 1:**
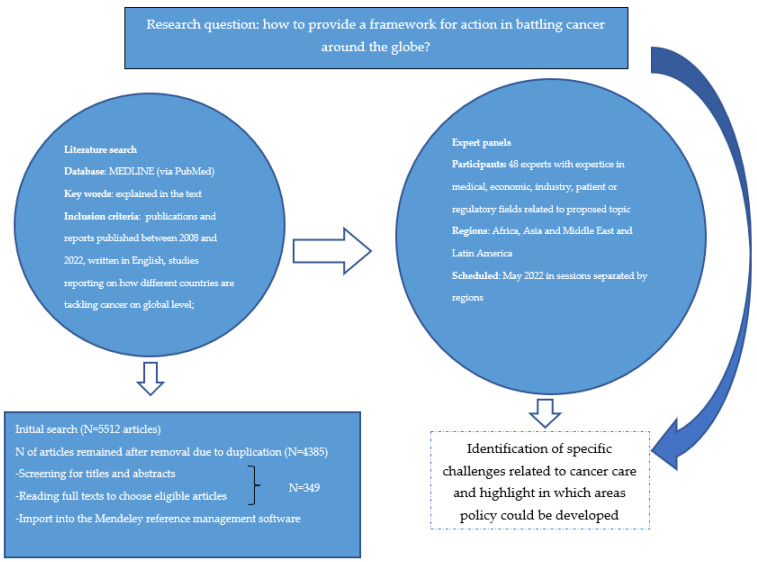
Research strategy applied. Methodology used to provide policy framework for tackling cancer on the global level.

The material was derived from extensive literature reviews augmented by a series of regional round tables organized by EAPM in the middle of 2022, with input from 48 experts. An initial literature search was performed to identify differences in cancer care in various global regions and also to identify different mechanisms in tackling cancer. A literature review was performed using MEDLINE (via PubMed) database with main keywords focusing on (synonyms of): ‘cancer care’ AND ‘globally’, ‘cancer care’ AND ‘Asia’, ‘cancer care’ AND ‘Africa’, ‘cancer care’ AND ‘Middle East’, ‘cancer care’ and ‘LATAM’, ‘cancer care’ AND ‘personalised medicine’, ‘NGS’ AND ‘Asia’, ‘NGS’ AND ‘Africa’, ‘NGS’ AND ‘Middle East’, ‘NGS’ AND ‘LATAM’, ‘cancer drug reimbursement’ AND ‘Asia’, ‘cancer drug reimbursement’ AND ‘Africa’, ‘cancer drug reimbursement’ AND ‘Middle East’, ‘cancer drug reimbursement’ AND ‘LATAM’, ‘molecular diagnostic’ AND ‘Asia’, ‘molecular diagnostic’ AND ‘Africa’, ‘molecular diagnostic’ AND ‘Middle East’, ‘molecular diagnostic’ AND ‘LATAM’. A total of 5512 articles were identified between 2008 and 2022 using PubMed database in the initial search. Inclusion criteria were: publications between 2008 and 2022, publications and reports written in English, studies reporting on how different countries are tackling cancer on the global level. After initial screening, 1127 articles were removed because of duplication. Searched articles were screened by titles and abstracts, and after that, full texts were read in order to choose eligible articles. A total of 349 articles were identified through the designed search strategy and imported into Mendeley reference management software. The relevant publications were used as a background for discussion in expert panels. A series of expert panels were organized, which gathered 48 experts from different regions, including Africa, Asia and the Middle East and Latin America (Figure 1, Table 1). Experts were chosen based on their expertise in medical, economic, industry, patient or regulatory fields related to the proposed topic. Regions, countries and experts were chosen based on their own research and published articles in the field and also their availability. Some regions such as Europe and North America were not included in this review since the aim was to obtain insight about the situation in other parts of the world where potentially less resources and funding are allocated for cancer care. Expert panels were held in May 2022 in separate sessions depending on the region, over the course of one week and chaired by Denis Horgan, Executive Director of the EAPM. Participants were asked to provide input based on their own experiences, and the aim of discussion was to highlight what policy framework could be developed to better tackle cancer globally. The format for all of the series was the same, and based on the expert panel discussions and literature review, certain conclusions emerged.

## 3. Results

Results are based on inputs and perspectives gained from experts on the organized panels as well as on data from literature search. They are grouped into two major categories. In the first, differences in cancer care are described in various world regions, looking in detail at individual countries’ perspectives. The second category focuses on different mechanisms needed in tackling cancer. 

### 3.1. The Regions’ Perspectives

#### 3.1.1. Asia

The experiences with cancer of countries in Asia are–like their responses–widely diverse (Table 2). Developing countries in Asia are facing growing cancer incidence, and according to WHO data, cancer is the second leading cause of death in the Asia-Pacific region after cardiovascular diseases [28,29]. Due to aging population but also lifestyle changes mainly connected with economic development, cancer has become a significant health problem there [30]. The number of people diagnosed with colorectal and lung cancer has exceeded that of Western countries. Reasons for that could be certain cancer-related lifestyle factors such as smoking, alcohol consumption, physical inactivity, obesity and high-fat, low-fiber diets adopted from Western parts of the world [28]. The Asian population has a high lung cancer burden, responsible for nearly 20% of cancer mortality, and it has the highest global rates of EGFR-mutated types [31]. Gastric cancer is prevalent in eastern Asia (e.g., Japan and Korea, which have the highest rates worldwide among both sexes) [32]. In Asia, the mortality-to-incidence ratio (MRI) is higher in the resource-limited areas (i.e., Mongolia, Tajikistan or Kyrgyzstan, MIR > 0.7). In the middle- and low-income countries, access to medicines is a prevalent issue. It is most severe in Bangladesh, Malaysia, Myanmar, Afghanistan and Nepal [31]. The Middle East region is facing increasing rates of cancer, with incidence predicted to double in the next two decades [33,34]. Most countries in the region lack national cancer registries. 

The continent is described as a “genetic goldmine” because of the diversity of its populations across its sub-regions, which both poses a challenge in developing appropriate tests and treatments, and at the same time presenting an opportunity to plunge further into new knowledge derived from the more neglected areas of cancer research in ethnically distinct subgroups. Many policy frameworks for PM implementation and government funding are emerging, with national plans and guidelines, but they are often marred by limited awareness among the general public and patients and limited education facilities. Poor health in these countries is mainly due to inadequate prevention and lack of prudent access to basic health care. Moreover, since pooling of risk and insurance is not in place, it has led to health-related impoverishment [14]. Higher rates of cancer incidence significantly influence the health and finances of individuals and states. A ranking of significant differences in readiness for personalized health care (measured by availability of health information, health services, personalized technologies, and the policy context) placed Singapore, Taiwan, Japan, and South Korea in the top positions, followed by Thailand, Malaysia, China, India, and Indonesia [35].

##### Malaysia

In Malaysia, tobacco and infections are reported as the principal causes of cancer deaths, which have a prevalence of close to 1/1000 [36,37]. Local experts argue for support for greater awareness and early diagnosis, particularly to enable remote communities to access equitable care with targeted therapies. They also want to see research featuring the characteristics of Asians and locals, linked to the use of community-appropriate risk score instruments. Additionally, they urge governments to take a more robust approach to pharmaceutical companies when negotiating prices: at present, nearly half of cancer patients experience financial catastrophe within a year of diagnosis.

##### The Philippines

In the Philippines, cancer was the second leading cause of death in 2020 [38]. Effective screening and prevention strategies exist for many cancers, but screening programs and public health education need more efforts [39]. There is a lack of health care professionals, particularly for servicing rural and remote regions, and a lack of infrastructure. Access to health care services is limited, and most care is paid for out of pocket [40]. In February 2019, the National Integrated Cancer Control Act was signed into law. The Cancer Control Law forms the National Integrated Cancer Control Council, which shall act as the policy making, planning and coordinating body on cancer control. This is attached to the Department of Health. The law mandates the creation of a Cancer Registry and Philippine cancer centers throughout the country. These shall aid government efforts to extend financial and other forms of assistance to impoverished cancer patients and to provide funds for cancer research. The Department of Health is also called on to intensify its cancer awareness campaign, provide the latest and evidence-based information for cancer prevention and treatment, and to supply sufficient access to cancer medicines. The law creates a cancer assistance fund. In addition, Philippine Health Insurance Corporation benefits for cancer patients will be expanded to include screening, detection, diagnosis, treatment, supportive care, rehabilitation, and palliative care. Overall, the Philippine Cancer Control Law has many goals in tackling cancer. It aims to ensure optimal cancer treatment and care for patients, as well as proper and affordable access to care, and improve the experience of cancer treatment and care but also support the recovery and reintegration into society of cancer survivors and eliminate different forms of burden on patients. A path to better cancer care was discussed in mid-2022 [41].

##### India

In India, two-thirds of health care needs are met by private health care, and more than two-thirds of health expenditure is out of pocket, with public health expenditure at <1.2% of GDP as one of the lowest in the world. The estimated prevalence of cancer is 2.5 million with an incidence 0.7 million cases per year. There are some 800,000 new cancer cases in India every year, with tobacco the most important identified cause of cancer, and India is one of the few developing countries that has created a National Cancer Programme envisaging control of tobacco [42]. Local experts express hope that reforms will strengthen primary health care and move towards universal health coverage featuring cancer care benefit packages, with a standards-based, interoperable, national digital health information system. Some strengthening of molecular and genomic testing facilities has been triggered by the COVID-19 pandemic, and there is a policy thrust to non-communicable disease control in general and cancer care in particular. There is a potential role for the thriving private sector as a driver of demand for and supply of new technologies, backed by assertive awareness campaigns that are gradually influencing policy decisions. The country lacks human resources and infrastructure to care for all patients, and solutions will depend on multisectoral intervention at the levels of health policy, public health expenditure, and capacity building in human resources, infrastructure and research. There are issues with long waiting lists, and also, some patients have to travel long distances to reach treatment facilities. There is a need to prepare an essential drug list for cancer chemotherapy, and chemotherapy services for common cancers must be available in all centers. The late stage at presentation is very often the main reason for the poor cancer survival rates in India, which are connected to the lack of diagnostic facilities in local hospitals [42].

##### China

China is like many other countries facing increased cancer rates. The top five commonly diagnosed cancer types in males were lung cancer (14.5%), prostate cancer (13.5%), colorectal cancer (10.9%), stomach cancer (7.2%) and liver cancer (6.3%) [43]. There are many investments in genome science and public health genomics-related programs and services. China has national genomics policies to address a variety of genetic issues, and also the infrastructure of public health genomics [44]. Many clinical resources provide a great foundation for the study of many diseases, including rare diseases, and research on biomarkers and big data are developing at a rapid pace within the country [45] (Table 2). 

##### Nepal

In Nepal, health care is underfunded. Because of this, the population suffers from financial risk in the case of using health services for diseases such as cancer. There is a partially implemented health insurance policy that has several limitations, is not available to everyone, and also lacks funding. All of this leads to delays in presentation, diagnosis and treatment [14] (Table 2).

##### United Arab Emirates

In the United Arab Emirates (UAE), the incidence of cancer has increased along with other non-communicable diseases. Therefore, a National Cancer Control Plan for 2022–2026 has been proposed that requires accurate data, a reliable cancer registry and periodic monitoring and evaluation. Cancer is the third leading cause of death in the UAE right after cardiovascular disease and trauma. Effective referral pathways are lacking, and access to diagnostic services is uneven. It is necessary to gather various stakeholders that will cooperate with the goal of successfully creating a policy framework. The government supports genomic projects, including the genomic and pharmacogenomic research of the Emirates Genome Program [46]; however, the demand for pharmacogenomic testing is not high enough in clinical practice. Better implementation will require clinical and basic research studies [47] (Table 2). 

##### Qatar

Projections are that the cancer incidence in Qatar will be tripled between 2010 and 2030 due to aging and population growth, with breast cancer (BC) as a leading pathology. BC screening in public spaces is being promoted. A National Cancer Research Strategy has transformed cancer care after benefiting from robust governance structures, committed leadership and wide multi-stakeholder involvement, and has been followed up by screening programs for bowel cancer and cervical cancer. Qatar has a National Cancer Registry, and there are plans to establish a National Cancer Information Centre. Cancer molecular genetic boards to integrate PM and genomics into cancer care are now in place. An integral part of cancer civil society in Qatar is supporting access to PM and genomic profiling, and advocacy about it among health care professionals and the community. Moreover, people living with cancer are aware of access to PM. The aims for the future are to include evidence-based approaches for public engagement, prevention and early detection, and especially the use of personalized approaches. Besides that, of great interest in Qatar are rare cancers, further local faculty development, and the evolution of Qatar’s high-impact cancer research portfolio. A companion document, the “Qatar National Cancer Research Strategy”, in 2014, provided a framework to develop basic, clinical, and translational cancer research while at the same time developing national capacity and capability with the use of the excellent existing academic infrastructure [48,49]. The Qatar Foundation is heavily involved in the development of precision medicine. In 2018, the Q-Chip was launched, the first genetic chip in Qatar that can store hundreds of thousands of gene variants in a device smaller than the size of a postage stamp [50]. In Qatar, there is the Qatar Biobank, an initiative created in collaboration with Hamad Medical Corporation and the Ministry of Public Health with the aim of enabling local scientists to conduct medical research on prevalent health problems in Qatar. The goal of Qatar Biobank is to enable scientists to conduct research on the most common diseases of today, such as cancer, among others. Qatar Biobank (QBB) collects information about the health and lifestyles of the Qatari population [51]. There is also the Qatar Genome Program (QGP), which is a national initiative that aims to map the genome of the local population [49]. In September 2022, the Precision Medicine and Functional Genomics Symposium (PMFG) was held in Qatar, bringing together various stakeholders to present the latest achievements and innovations in biomedical research and ways to translate them into precision medicine solutions [52] (Table 2). 

##### Lebanon

In Lebanon, cancer rates are increasing and consequently the burden of cancer cost. Patients have access to treatment in public and private hospitals, and the Ministry of Public Health and third-party payers are providing the financial coverage. Cancer drugs are free of charge for uninsured patients, and this country has one of the most developed health care systems in the region. Hospital infrastructure is adequate, and novel equipment is available, as well as physicians and health care professionals. Moreover, many programs have been put in place to raise awareness about cancer screening and prevention through educating and counseling the population, and cancer research has been established by the health ministry [53].

##### Kingdom of Saudi Arabia

Oncology care is provided through the public sector, and all cancer care facilities are freely accessible to citizens. There are cancer screening programs for breast cancer, colorectal cancer and cervical cancer. There is a significant deficiency in the native oncology workforce and expertise, resulting in out-of-the-country recruitment [54,55,56].

The difference across the wider Australasia is marked between individual countries. High-income countries such as Australia offer universal health care and strongly funded research [57], high levels of public trust in health care governance and longstanding policies on personalized or precision medicine, leading to wide take-up of genomics, biomarkers, smart data generation and analytics, all bolstered by substantial national dialogue at the academic, industry and public levels. Japan, too, has established centralized databases (and reimbursement) for clinical and genomic tests, and outcome data that provide active feedback on treatment options [58], and the system is characterized by strong leadership and collaboration among academia, industry, government, and patients, with secure data transfer, strong links to hospitals, and good provision of education for health care workers, politicians, patients, and society, so personalized medicine in cancer is rapidly progressing. In Korea, national health insurance covers more than 70% of medical costs and provides NGS tests for cancer and rare genetic disorders [59]. Information technology infrastructure is well-developed with the wide use of EHR big data platforms in major hospitals. The government supports and controls research through to clinical implementation and commercial utilization, permitting pseudonymized personal data use for commercial and public health purposes, and the health care industry is growing [59]. 

#### 3.1.2. Africa

People from Africa are very adversely affected by cancer, with 1 million new cases per year, and a projected 70% rise in new cases by 2030 [60]. African populations exhibit huge genetic diversity, and better coverage will require work with a wider range of ethnic groups to decode the true genomic imprint that influences health [61,62]. But the late entry of Africa into genomics research and the cost of genome characterization hamper advances. Sustainable governance in pharmacogenomics needs to be established. The challenges are intensified by lack of awareness, late diagnosis and lack of effective treatment protocols. The problems are compounded by a susceptibility to aggressive disease that is poorly elucidated owing to low engagement in clinical trials. On top of that, other problems include continuing reliance on traditional therapies, a scarcity of medical oncologists, lack of adequate health facilities, more extended hospital waiting hours, and high treatment costs. The principal causes of poor health in poorer countries are inadequate prevention and lack of reasonable access to basic health care, together with health-related impoverishment resulting from a lack of risk pooling and insurance [63,64,65,66]. The health and finances of individuals and states are highly influenced by the increasing prevalence of cancer [14]. In Zimbabwe, 80% of cervical cancer patients present with advanced disease [67], and in Tanzania, more than 90% of breast cancer patients were diagnosed at stage III and above [68]. Factors such as limited knowledge of signs and symptoms of cancer, limited screening facilities, and fear of surgery are some of the major barriers to early presentation and cancer diagnosis [61].

Wide disparities in the size of countries in the region, in their wealth and in a score of other local characteristics make many generalizations difficult. The Africa Union Development Agency has ambitions to promote genomics and PM in concert with EU aid, and its vision embraces identifying the germline and somatic alterations at the origin of cancer development, exploring molecular and histological biomarkers behind resistance to current targeted cancer therapies, and promoting early diagnosis and better care for patients and their families through precision oncology implementation [69].

##### Tunisia

Tunisia runs human genome programs, and the Oncogenetics Unit at the Institut Pasteur in Tunis is conducting research-based genetic diagnosis. This is linked to a regional grouping in North Africa that has set a goal of implementing PM and is already running a pilot project on lung cancer that covers data generation through to translation in clinical settings, accompanied by training and recommendations for health authorities. PerMediNA-Precision Medicine in North Africa is linking the Instituts Pasteur in Tunisia, Algeria, Morocco and Paris with EUR 1 million funding from the French government. The intention is to co-create health and research agendas based on local needs, with investment in African institutional capacity building, leadership, and ownership, in alignment with existing bodies. Challenges include generating awareness of the value of PM, empowering patients, reducing patient payments for testing and drugs, developing infrastructure and HR for tests and sequencing and genotyping facilities along with biobanks, promoting the wider use of EHR, and initiating local clinical trials, as well as the creation of new profiles, e.g., DPOs, bioinformaticians, molecular biologists, etc. North Africa has seen some interesting procedures aimed at easing patients’ access to medicines through price controls (Table 3). 

##### Angola

Angola’s government has shown willingness to develop genomic surveillance activities in response to the high burden of infectious diseases in the country, with staff and centers based on existing surveillance programs. However, a national plan will be needed to effectively mobilize resources, train staff and reinforce laboratory biosafety measures. The Ministry of Health plans to have a national genomic surveillance center and lab from 2022, in collaboration with WHO.

##### South Africa

South Africa, with its high burden of NCDs and HIV, has established funding for PM, with a genome program and a PM think tank, aiming at a research strategy and product pipeline with an NCD focus on cancer. It has to cope, however, with less than ideal local conditions, including a technology gap, lack of population-level genomic data, barriers to conducting well-powered studies, unresolved ethical and legal issues, lack of access to targeted therapies, and high costs. PM has not yet been incorporated into legislation, reimbursement is not yet a norm and currently causes inequalities, and infrastructure lacks critical mass and standardization. Evidence must be mobilized to win support for more innovative solutions, especially in oncology. However, some clinical care guidelines are now being developed in oncology, and private care is moving toward a patient-centric approach, while government is moving toward universal health care coverage and reinforcing its research agenda. The PAN African Cancer Research Institute is the first cancer-based center to offer precision oncology to the public health sector, and other cancer centers of excellence are also driving precision oncology. A key need now is to foster international collaboration to fast-track the country’s development in PM.

##### Cameroon

Cameroon has a national strategic plan for prevention and cancer control, but it makes no reference to personalized medicine, genomics, or biomarkers. Governance and the political will to push PM are lacking, and funding is limited. So, combined with low patient awareness and education, uptake is low, mortality is high (particularly in cervical screening and HPV), breast cancer (BC) genetic testing is out of reach for most patients, and targeted therapies are not available locally. On top of that, there is a lack of trained workforce and infrastructure, ethical bodies are not experienced with PM studies, and there are unresolved issues on data sharing and on validation and regulatory approval of genetic tests and targeted therapies. While demand for PM in oncology is growing, it is not prioritized in health strategy and requires further investment from both the public and private sectors and attention from policy makers (Table 3).

##### Kenya

Kenya has set universal health coverage as a priority, with novel health care delivery and public health systems supported by modern technologies and the promotion of healthier lifestyles, along with new technologies to enhance disease surveillance, prevention, early diagnosis and treatment. However, moves toward a strengthened social health protection schemes and policy reforms to improve access are restrained by the high cost of health care, poor diagnosis and the emergence of infectious and non-communicable diseases, further disrupted by COVID 19. Thirty-five percent of Kenyan stakeholders responded to a survey in positive terms about awareness of PM, but there is a recognized need for increased stakeholder engagement to boost awareness, skills, ethical/legal frameworks and infrastructure [70] (Table 3).

##### Nigeria

In Nigeria, around 100,000 new cases of cancer occur every year [71]. Regarding the financial aspect of health care, Nigeria spent only around 0.5% of its 2017 budget. There is a lack of funding, inadequate prevention, lack of proper access to basic health care, as well as health-related impoverishment [72]. Other challenges are that the Nigerian public health sector still struggles to integrate clinical systems into research, physicians are often resistant, patients are unaware of novel drugs, and there is a lack of immediate availability of novel drugs [14] (Table 3). 

#### 3.1.3. LATAM Perspective

The cancer incidence in Latin America (LATAM) is low, but mortality is high, and forecasts are for a 35% increase in cases by 2030 in South America and 42% in Mexico [73]. In 2020, the region saw more than 1.4 million new cases and more than 600,000 deaths. BC accounts for 15% of cases, prostate cancer for 14%, colorectal cancer for 9%, lung cancer for 7%, and stomach cancer for 5% [74,75]. The challenges that are present in this region range from inefficient care delivery and slow acceptance of improvements to the fact that patients have high out-of-pocket costs (from 16% in Uruguay to 43% in Ecuador) [76]. Additionally, there are not enough reliable cancer registries, and investments in cancer drug development and research are low. Around 40% of the LATAM population lives in rural areas with restricted access to health care support [77], and populations are still excluded from social security and public financing. After Brazil, Chile has the highest public funding for clinical studies, followed by Argentina. Bolivia, Paraguay and Uruguay provide very little [73,74]. Income and level of education are highly correlated with cancer mortality in Latin American countries, even in the region, and organized screening programs are not in place. Although there have been some improvements regarding socioeconomic disparities, cancer mortality has not decreased substantially in the region. Moreover, access to cancer screening and treatment is also highly associated with socioeconomic inequalities, income and education level. In most LATAM countries, only opportunistic screening for cervical and breast cancers is available, while lung cancer is still not a priority for doctors there. The use of tobacco is a leading cause of cancer [74], and not many people are aware of screening options. It is of most importance to establish cancer registries across all countries that would contribute to a public cancer policy that includes screening programs [77]. Policy makers should invest more in sustainable cancer control programs and initiatives that aim to increase awareness and engagement in prevention and screening. Such investments could bring better outcomes at the population level [74].

##### Brazil

By some estimates, there were 625,000 new cancer cases in Brazil in 2020. The cancer care network in Brazil, in both the public and private sectors, from prevention to palliative care, includes primary care, home care, and specialized outpatient and hospital care, in addition to support systems, regulations, logistics, and governance [78]. With the establishment of the National Health System (SUS), Brazil has been publishing an array of rules to ensure the access of individuals to full care and reduce mortality and cancer-caused impairments, diminish the incidence of certain types of neoplasms and contribute to ameliorating the quality of life of cancer survivors. SUS subsidizes cancer treatment for approximately 75% of the population. Brazil’s non-communicable diseases (NCDs) plan, which runs from 2011 to 2022, includes cancer. It also has a uterine cancer plan that has been running since 2010. 

New laws addressing attention to cancer came forward between November 2021 and March 2022 to attempt to redirect and reinforce control initiatives [79]. In Brazil, the primary care network is the patient’s entryway to the health system, playing a decisive role in actions to promote health and prevent and track cancer. If cancer is suspected, the patient seen in the primary care unit is referred to secondary care for further investigation [78]. Cancer treatment is performed in specialized care units, including high-complexity oncology centers (Centros de Assistência de Alta Complexidade em Oncologia–CACONs), which treat all cancers, including hematological cancers, and may or may not treat pediatric cancers; high-complexity oncology units (Unidades de Assistência de Alta Complexidade em Oncologia–UNACONs), which treat the most prevalent cancers with or without radiotherapy, hematology–oncology, and/or pediatric cancer services; and hospital complexes [79].

The data on the mapping, number of patients, and quality of cancer care is lacking in Brazil which poses a challenge in the creation of evidence-based policies in Brazil. What often is a case is that different cancer treatments are concentrated in single health care units in the country. Access to cancer treatment highly depends on the concentration of services in large urban centers and also on the fact that some patients have to travel long distances to receive proper treatment. Taken into account the territorial extent of Brazil, it is even more important and challenging to assure a highly coordinated multi-layered health care system [79,80].

In the first national cancer policy from 2005 in Brazil, the “need to structure a regionalized and hierarchical service network that guarantees comprehensive care to the population” was emphasized. Specialized cancer care centers in Brazil are lacking, and also, the question is to what extent municipalities with centers of high complexity can serve their resident populations. A study from 2022 showed that more than half of cancer patients have to travel to receive treatment; regional disparities are present and last for a long time, thus hindering the accessibility of patients from certain parts of Brazil; there is a need to better balance the supply and demand sides since the hubs and attraction poles are concentrated in only a few municipalities in certain regions [78]. Brazil ranks very well in working to reduce smoking rates as a cancer prevention strategy [81]. To increase access to NGS in oncology, in Brazil, agencies and payers will be required to collaborate in building data collection infrastructure. Moreover, novel pricing and payment approaches will be needed, based on a comprehensive assessment that takes into account the potential acceleration of patient access and PM’s contribution to managing uncertainty and budgets. However, post-market collection, review and reporting of therapeutic performance will be needed to update reimbursement status, pricing decisions and clinical guidelines or support a disinvestment decision if the value cannot be demonstrated [82] (Table 4). 

##### Colombia

According to data from Globocan, Colombia had an age-standardized rate (ASR) of 178.8 new cases of cancer per 100,000 people in 2018 [83]. Projections are that there will be 148,600 new cases of cancer annually by 2030 and 189,988 new cases by 2040. Colombia has a National Cancer Control Plan (Plan Decanal para el Control del Cáncer de Colombia 2012–2021, or PDCCC), which sets objectives informed by a series of national and international standards and regulations and aims to: emphasize cancer prevention; improve early detection; improve quality of cancer care and recovery of cancer patients and survivors; strengthen national information systems; and improve the training and development of practitioners. A common challenge, according to a survey from 38 stakeholders, was the fragmentation within the health system, resulting in inequitable health outcomes, costs, and quality of services between the public and private systems and among geographic regions. Other issues included: inconsistent enforcement of the regulatory frameworks related to cancer prevention, control and care, high costs of cancer services, lack of transparency in decisions, and inconsistent levels and quality of services across the country [84]. Oncology services are limited, which is aggravated by ill-timed access and lack of continued care. Many Colombian institutions provide oncological care only for some specialized services (e.g., only radiotherapy) [85], which leads to delays and fragmentation in the treatment of patients who need treatment combinations. Access to cancer treatment is widely influenced by the concentration of services in large urban centers and the consequent large distances traveled by patients [78,86] (Table 4).

##### Mexico

In Mexico, as in other developing countries, cancer is the third leading mortality cause. The most common types of cancer among Mexican men are prostate, colorectal, lung, gastric, and testicular cancer at younger ages. Among Mexican women, they are breast, uterine cervix, and colorectal cancer. In 2008, a general law was approved for the control of tobacco, and in 2009, in compliance with the General Law Regulation for Control of Tobacco, pictographs and warnings were implemented on the packages. Still, a small part of the population in Mexico has access to genetic analyses to identify factors associated with the development of some types of cancer, for the early detection of a tumor, or to take the option of chemotherapy or prophylactic surgery, which economically would be more affordable. Nevertheless, these services are not in high demand, nor are any significant government genetic counseling programs up today. Genetic counseling and molecular diagnosis are routinely offered by family cancer clinics in a few level 3 government and specialized private hospitals [87]. It is becoming more common that these are subsidized by big pharma as a means to promote the prescription of their drugs for targeted therapy. In Mexico, Seguro Popular (SP) was created to provide universal health coverage, including cancer care. SP’s reimbursement guidelines were set to cover medicines for basic cancer care and define reference prices, which are similar to international reference prices. Eight percent of SP’s resources were allocated to “Fondo de Protrección contra Gastos Catastróficos” (FPGC), of which 28% finances cancer care [88]. Unfortunately, with the new government taking office in 2018, SP was supposed to be replaced by the new Institute of Health for Wellness (Instituto de Salud para el Bienestar or INSABI), which has faced enormous complications in its proper operation, leading, among other calamities, to the crisis of scarcity of medicines to treat infantile cancers in particular, and of oncology treatments for the entire population in general. Unfortunately, trying to fight the corruption alleged to be the cause of overpriced medicines, acquiring abroad directly from manufacturers and centralizing their distribution for the entire country, has resulted in an enormous shortage of many drugs for cancer and several other medical conditions. In Mexico, citizens have access to a wide range of special cancer diagnostic preventive measures, such as Pap smears offered for women aged 25 to 34 every three years. Due to the large proportion of the population that smokes, the incidence of lung cancer is also high, especially among men [89]. Moreover, diet and cancer incidence are highly correlated in Mexico. Due to poor nutritional education and an abundance of cheap, high-calorie unhealthy foods, obesity is a big problem in Mexico. What has so far shown certain positive results is the introduction of the tax on tobacco smoking and sugar-sweetened beverages. Regarding financial coverage, about 50–60% of cancer patients in Mexico are fully covered. The new President has shown signs of desperation to implement the INSABI mission and to keep his campaign promise of giving the population free access to treatments and medicines in the government hospitals. However, these are severely underfunded, and thus, a vicious cycle precludes equaling or exceeding what SP had achieved (Table 4).

##### Chile

The cancer incidence in Chile is substantial, but also many health-related disparities influence distribution of the cancer burden and the quality of cancer care. Cancer is the second leading cause of death and accounts for 23.4% of all deaths in the country. Most common are prostate, colon, breast, stomach, and lung cancers. Chile has witnessed a significant reduction in cancer-related mortality, by 5.2% in women and 5.5% in men. This reduction, noted in the period from 2015 to 2019, is greater than the reductions in other countries such as Brazil, Mexico, and Colombia. Health-care Chilean network is mainly responsible for promoting cancer prevention strategies. Access to the primary health-care system is universal and free in Chile. Childhood undernutrition, infectious diseases, and respiratory diseases have been addressed very well by this policy. However, it did not really succeed in implementing national programs for cancer prevention. Currently, there is no national registry of cancer in Chile, although there are five population-based provincial registries. However, none of them operate in the metropolitan region of Santiago, which covers almost half of the country’s population. A national pediatric tumors registry exists. Well-trained surgical oncologists are lacking, in addition to a shortage of other oncologists. The shortage of equipment is also present, which is highlighted by the number of linear accelerators (the country could use 80 linear accelerators, but it currently has only 40), and also, major cancer survivorship programs are lacking. It will be of great importance to provide these services as the number of cancer survivors increases [90].

Chile has radiotherapy coverage of more than 100%, which means that more than the estimated proportion of patients requiring radiotherapy are able to access it. The country has invested in preventive measures such as Pap screening tests for women aged 25–64 every three years and an HPV vaccination program that operates as part of a routine immunization program. It also has a program to screen those over 40 with a family history of stomach cancer and a current ulcer and is piloting a screening program for colorectal cancer. As in many countries, Chile has high rates of prostate cancer in men, and for women, breast cancer is most common [89] (Table 4). 

##### Peru

The lack of local genomic laboratories means samples requiring comprehensive genomic analysis have to be sent abroad, and universities remain little engaged in innovation in molecular tests. Peru was, however, a participant in the clinical validation of the 21-gene recurrence score. Technology and reagents for NGS are three times more expensive than in the US, and most tests (and targeted treatments) are not reimbursed. Strong loyalty strategies among foreign laboratories impede local competition, and skills are scarce in wet labs and bioinformatics. There is little medical familiarity with biomarkers and genetic tests, highlighting the need for training and public education programs [91]. However, a governmental cancer control program and development of a national tumor bank are underway, and oncologists are highly trained, increasing cancer research. There is a need for more provision and reimbursement of liquid biopsy (LB) in LC, and more funding of studies of the genomics of cancer in the highly diverse Peruvian population. Strategies are needed to lower prices for targeted drugs and genomic tests (Table 4). 

##### Venezuela

Overall health care spending is around 5% of GDP. Governance of PM in oncology is only modest, with low availability or implementation of multidisciplinary guidelines, and no national cancer institute or plan. Privacy and cybersecurity continue to suffer from the absence of a legal framework for patient privacy and data protection. Greater efforts are needed in adoption and awareness of PM, and availability of education, training and outreach activities is low. However, infrastructure in terms of data collection and biobank organization is improving, and reimbursement policies are being implemented [92] (Table 4). 

## 4. The Tools and Conditions for the Job

The different performances from country to country and region to region are reflected in the use they make of the tools increasingly available to bring personalized and advanced care into oncology. This section compares and contrasts some of these experiences across the range of key factors.

### 4.1. Uptake of Molecular Diagnostics

NGS testing in clinical care is growing in every major region [93], but the picture is heterogenous in Asia, among Indonesia, Malaysia, Singapore and Thailand [94], and in Africa, where many countries face challenges [93]. Access to advanced diagnostics needs to be designed and implemented in a patient-centric, rather than institution-centric, manner, as a global meeting on molecular diagnostics organized by WHO and the African Society for Laboratory Medicine focused on in discussing the diagnostic integration of infectious disease molecular testing. Sharing technologies across programs is the first step in diagnostic integration. It then drives the integration of additional laboratory services and structures. Development of a holistic integrated system across diseases will benefit cancer programs as well [95].

The evolution has been facilitated by the Human Hereditary and Health in Africa (H3Africa) initiative. This initiative aims to promote a contemporary research approach to the study of genomics and environmental determinants of common diseases in African populations. Genomics activities in Africa are currently being carried out mostly in the context of research. More efforts need to be made to implement genomics in health care settings. Besides that, there is a need to bring research and health care closer together and to promote the translation of basic research into health care systems. Recommendations exist about proper infrastructure requirements for genomic medicine implementation, how to implement genetic testing, how to use clinical data, and how to translate results into clinical practice, etc. Most of the technologies used for detecting genetic mutations and alterations are developed using non-African populations [61]. With support from H3Africa, infrastructures for genomics research have been set up in several locations across Africa. Maintaining the newly established infrastructure lies with the research stakeholders, particularly the African scientists. The Network for Genomic Surveillance in South Africa (NGS-SA) is a network of laboratories, scientists and academic institutions who rapidly respond to public health threats. Different hospitals and universities contain well-established laboratories. Although Africa has an uneven burden of diseases, it has not been part of many large-scale genomics studies, as well as clinical trials. In order to improve the precision and accuracy of health care decisions, African countries need to generate new knowledge on the genetic landscape and take ownership of that.

There are big differences in the approach to molecular testing in LATAM countries. Some of the existing challenges are: difficulties related to NGS interpretation and lack of trained personnel, bioinformatic support, and molecular tumor panel networks [91]. Government and the private health sector could work together with molecular diagnostics companies and the pharmaceutical industry to increase access to comprehensive genomic profiling. Diagnostic–therapeutic strategies for each population in LATAM countries are needed, taking into consideration the relationship between ethnicity and types of biomarkers in each population. Cancer genomics resources and specialized personnel are scarce—Brazil and Mexico have the highest numbers of NGS platforms, while Peru and Ecuador have the lowest [75]. A support program from Brazil has seven pharmaceutical companies working together to provide timely and accurate molecular diagnosis of advanced lung adenocarcinomas both in the public and private health care systems [91]. 

The molecular diagnostics market is growing rapidly in the Asian region and is projected to reach USD 1017.7 million by 2031 [96]. In Korea and Japan, NGS has been adopted as part of clinical practice, especially in patients with gastric cancer [32].

### 4.2. Uptake of Biomarkers

The benefits of biomarkers need to be extended to health systems in developing countries, despite the barriers of cost and inadequate infrastructure. A lack of data in diverse populations impedes wider application of biomarker-based technologies, highlighting the risk that biomarker-based products or tests could be less efficacious or even have an increased risk of toxicity in other populations if they are developed using genomic data from only one population. The Pharmacogenetics for Every Nation Initiative is helping countries to develop the education, guidelines and infrastructure necessary to lay the groundwork for personalized medicine. It screened populations in 104 countries for known biomarkers in genes involved in different processes such as drug metabolism, transport, and also target genes for the medicines on the World Health Organization “Essential Medicines List” [97]. 

The use of biomarkers is not yet widely practiced in Latin American countries. Because of the current financial and technical limitations associated with insecure health care systems, as well as inadequate access by low-income people to quality health care, such tools are not yet fully affordable. Due to the genetic admixture in the populations of Latin America, there could be a difference in responses to treatment from one population to another. It is necessary to evaluate and conduct a deeper analysis in different populations of Latin America with the aim of finding the best biomarkers and therapeutic options for each population. Physicians and all care providers involved in cancer screening, diagnosis, and treatment need up-to-date training on how to integrate genomic and molecular data into clinical practice [73].

To gain insight into population-specific biomarkers for early diagnosis and follow-up, ad hoc designed clinical studies in African populations are needed. Various factors such as pathogens, carcinogens, dietary habits, etc. can influence tumorigenesis depending on the population and geographical setting [98]. It is evident that the incidence of cancers associated with infectious pathogens is higher in Africa and Asia than in other continents. Certain forms of cancer, such as prostate cancer, are more aggressive in the African population. Therefore, more African-based studies are needed to confirm the applicability of circulating biomarkers and LB technologies in cancer diagnosis and treatment in Africa. African studies began in the late 2000s, and most were conducted in Egypt. Only a few studies have been conducted in other countries, including Tunisia, South Africa, Gambia, Cameroon, and Senegal [99].

The uptake of precision oncology across the APAC region is uneven mostly due to the presence of different health care systems and other health care priorities. Furthermore, growing collaborations and partnerships between government organizations and pharmaceutical companies for the development of biomarkers are expected to boost the growth of this segment. In 2016, ESMO published guidelines that take into account the ethnic differences associated with the treatment of metastatic NSCLC cancer in Asian patients [39]. 

### 4.3. Uptake of Liquid Biopsy (LB)

The global LB market is constantly growing and is expected to reach USD 5 billion by 2030. Some projections are that the Middle East LB market will reach USD 175.2 million by 2032, after it was valued at USD 43.7 million in 2021. Due to the increase in the geriatric population in the Asia-Pacific region, it is expected that the market for LB will further grow. This region is contributing substantially to the global LB market, where China and India are the major contributors [100].

In Singapore, researchers at the National University of Singapore announced they are on the verge of having a blood test capable of keeping track of any cancer’s development. They conducted the Oncotarget study using samples from patients with bowel cancer, which is Singapore’s most common type of the disease. One of the major problems they faced, as one of the authors of the published study said, was that it was hard to find the circulating tumor cells (CTCs) they used for their procedure. However, after the difficult first step of isolating and collecting the CTCs, the team was able to identify two specific mutations associated with that type of cancer, the study said [101].

In China, regulators have approved Amoy Diagnostics’ EGFR mutation LB. Traditionally, doctors identified the non-small cell lung cancer patients most likely to respond to EGFR inhibitors such as Iressa and Tarceva by taking and analyzing tumor samples. The invasiveness of this procedure—and inability to obtain samples from some patients—led test makers to work on diagnostics capable of identifying EGFR gene mutations in plasma samples through the detection of circulating tumor DNA. CFDA opened the door to the use of blood-based diagnostics in the identification of patients with the EGFR mutation in 2015, when it revised the label for gefitinib, an EGFR inhibitor sold by AstraZeneca as Iressa. The updated label cleared doctors to identify the mutation in blood samples when they are unable to access tissues for analysis. The approval of such a test comes 18 months after the U.S. FDA cleared an LB for use for the first time. That earlier approval involved another EGFR diagnostic, Roche’s cobas EGFR Mutation Test [102]. 

The Latin American LB market is growing, which is mostly due to the increasing number of infectious diseases and also because of an increased focus on developing new technologies in the health care domain. Factors further contributing to the growth rate of the LB market in this region are the favorable reimbursement policies and the increasing awareness of the use of precision medicine. There has been a significant increase in demand for tests and services attributed to increased demand for non-invasive diagnostic services for treatment selection and monitoring. In addition, there are more and more molecular diagnostic companies that provide LB tests.

Some of the companies operating in the LB market in Latin America are Janssen Diagnostics, Qiagen, SRI International, Natera, Sysmex Inostics, etc. The market demand for LB is growing in Mexico, as cancer is the third leading cause of death there. The Brazilian market is geographically the largest contributor to the Latin American LB market [103].

In order to identify population-specific cancer biomarkers in LB, adapted and optimized for African countries, epigenetic variations, host genetics, and tumor genetics need to be investigated and taken into account. In addition, it may be challenging to determine the sensitivity, specificity, and efficacy of LB methods in African patients, given that most available products have not been tested in African populations. For this technology to emerge and advance in Africa, it is necessary to build large capacities in terms of specialized staff training and infrastructure. This is not only relevant in the domain of molecular biology and clinical research, but also in computational biology and bioinformatics, since the goal is to standardize and validate potential biomarkers. LB approaches would promote the practice of evidence-based precision medicine in Africa, applying therapies when needed, and avoiding the human and societal costs of under- and over-treatment. Kenya recently became the third African country, after South Africa and Tunisia, in which the use of LB is commercially made available to the public. The estimated cost of each test was considered to be seventy thousand Kenyan Shillings (about USD 7000), which is practically unaffordable to middle- and low-income earners that form the majority of the country’s population [99].

### 4.4. Uptake of Real-World Evidence (RWE)

The opportunities offered by real-world data (RWD) are not immediately easy to seize. Real-world evidence (RWE) intended for patients, clinicians, payers, and regulatory decision-makers [104] needs to evolve alongside the development of precision oncology and overcome technical and logistical barriers including limitations in cross-study data comparability, heterogeneity in data storage, collection and representation, and difficulties in the storage and transfer of large datasets [105]. It can play a role in marketing authorization, early access schemes, and pharmacovigilance, in HTA decision making, in academic research, and in health care management and financing. However, ethical, regulatory, and legal issues need to be resolved to achieve broad patient consent and data sharing. Consensus guidelines are needed to standardize the methodology of RWE-based studies and best practices for data sharing in line with data-protection regulations. It is important to build infrastructure that brings data together and that invests in future analyses [22]. A successful application of RWE requires well-defined research questions and an appropriate study design. Appropriate RWD sources and validated data extraction and measurement procedures are also essential. When interpreting the results, the limitations and totality of the evidence must be taken into account.

There are many challenges regarding the uptake of RWE in Africa. Some of the factors that prevent data from realizing its full potential in the health sector of Africa are the lack of data storage infrastructure, ineffective health data management, shortages of trained health professionals, lack of financial resources for health, and poor implementation of services [106].

Because of the history of slavery, biobanking African samples out of Africa has been a sensitive issue at the participant as well as state level. Nonetheless, a recent qualitative study in Nigeria showed that when participants understand the purpose of biobanking, they believe it is beneficial and are willing to share their samples with other researchers [63].

Currently, Asia is missing the framework for RWE. Asians are often underrepresented in clinical trials unless the research is conducted directly on the Asian population. RWD can help fill the evidence gap. Additionally, in several Asian health care systems, reimbursement decisions are not made at market entry. This would enable the collection of RWD/RWE with the aim of greater certainty about the effectiveness of technologies in the local environment and information about their appropriate use. Unlike the USA and Europe, Asia often relies on clinical effectiveness data from non-clinical trial sources (observational studies or disease registries, for example) for regulatory and reimbursement purposes. This is why RWD are of particular importance. Only about 17% of clinical trials are conducted in Asia due to barriers related to financial and human capacity, ethical and regulatory systems, lack of research environment, and operational issues. The Asian population may be underrepresented in key RCT trials. These considerations are crucial because medical treatments must reflect biological variation, such as differences in body weight or pharmacokinetics and/or pharmacodynamics due to different genetic makeups between Caucasians and Asians, and nonbiological variation—for example, differences in local clinical practice guidelines driven by constraints on budget and resources [107].

In China, the China Real World Data and Studies Alliance (ChinaREAL) has issued five guidance documents on how to design observational studies, how to develop research databases using existing health and medical data, how to develop patient registries, how to conduct PrCT, and how to appropriately analyze RWD. In January 2020, the National Medical Products Administration in China issued guidance on the use of RWE for drug development and assessment for pilot testing. In Singapore, while the existing HTA guidance documents describe the circumstances under which clinical effectiveness data from sources other than clinical trials may be used as supplementary evidence to inform decision making, most reimbursement decisions are predominantly informed by RCTs. In Malaysia, RWD are considered a priority area of pharmacy research to monitor therapeutic outcomes. However, there is currently no specific plan to develop guidelines on how to use these data for HTA. The PHL National Formulary System Guidelines (AO 2016-0034) in the Philippines do not specifically state whether there is a need to collect RWD/RWE. In China, RWD are collected from regional EMRs, EMRs from individual care facilities, disease registries, and claims databases. In order to ensure the quality of RWD, the HTA agency in China only accepts clinical efficacy data collected by teaching hospitals or used in published articles in peer-reviewed journals. In Taiwan, an analysis of the cost-effectiveness of Alzheimer’s disease treatment was conducted. Transition probabilities derived from observational data from a clinical sample, cost data from the National Health Insurance Database, and a caregiver survey were used. In Bhutan, to estimate health care utilization (outpatient visits and hospitalization) related to a specific viral infection for vaccine evaluation, a combination of data from the Health Management Information System (HMIS) and Royal Centre for Disease Control (RCDC) surveillance was used. Use of RWD/RWE in HTA and decision making varies across Asian health systems, with some using it primarily to inform pharmacoeconomics, pharmacovigilance, or pharmacoepidemiology. Others also use it for reassessments, where RWE informs price adjustments and/or disinvestment decisions pertaining to the technologies that have gained an initial positive reimbursement decision. 

Although RWE use is emerging across the decision-making process worldwide, and its application is becoming widely accepted by regulators and payers, the uptake of RWE in LATAM is not as common as in the US and EU, and significant gaps are being seen in the health care system, compared to other regions in the world [108]. Poor data quality, unclear understanding of RWE applications, and lack of clear policies and procedures on RWD access and protection were some of the frequent barriers faced in the collection and use of RWD [108]. Key challenges of data integrity, quality, and security are still present, and there are gaps in skilled personnel, a lack of confidence in observational research and lack of trust between users and data holders. Arguments have been made for a central authority to steward health information systems to ensure interoperability of and quality guidelines for RWE.

### 4.5. Reimbursement and Other Regulatory Issues

In certain Asian health systems (e.g., China, India, Indonesia, Malaysia, the Philippines, Singapore, and Thailand), it takes several years after market entry for reimbursement decisions to be made. A study conducted by ESMO showed a lack of availability of essential medicines in some countries on the WHO Model List of Essential Medicines, such as oxaliplatin and capecitabine—which are the backbone of colon cancer treatment in both the curative and advanced settings [31].

Patient safety can be compromised by counterfeit or low-quality complementary medicines obtained from unregulated sources. Therefore, there is a growing need to establish and regulate the provision of complementary therapies with proven safety and benefits as adjuncts to conventional cancer care. Vietnam and the Philippines face exceptional economic difficulties regarding the use of complementary medicine. In Myanmar, expenditure on complementary medicine, relative to no such expenditure, is consistently associated with a lower risk of adverse financial outcomes. Economically disadvantaged households that reported out-of-pocket spending on complementary medicine were more vulnerable to adverse financial outcomes, regardless of country of origin.

In Kuwait, Lebanon, Qatar, Saudi Arabia, and the UAE, tendering is used as a mechanism for the procurement of generic pharmaceuticals. In Kuwait, Saudi Arabia and the UAE, generics are reimbursed according to the same system as off-patent pharmaceuticals. In Lebanon, tendering and formulary management are dominant in generic procurement, but in Qatar, reimbursement is achieved through a combination of different measures, involving tendering, formulary management and IRP. Support provided to local manufacturers includes predominantly discriminatory reimbursement practices favoring local over multinational manufacturers (seen in Bahrain, Jordan, Kuwait, Lebanon, Qatar, and Saudi Arabia), and generic pricing policies to encourage local production and provide more beneficial pricing arrangements to local over imported generics [109]. In Jordan, public sector procurement is performed through annual tenders issued in the generic name of the pharmaceuticals or therapeutic groups jointly issued by four governmental parties. The system was introduced to prevent different public health institutions from buying the same pharmaceutical at different prices, which was found to lead to shortages and higher pharmaceutical spending. Adjustments could be made elsewhere to increase generic procurement for the public sector. The Lalla Salma Foundation in Morocco improved access to innovative pharmaceuticals for poor patients via a memorandum with pharmaceutical manufacturers. The Lebanese government reduced the prices of 30 branded and 60 generic pharmaceuticals to increase access (though, despite these price reductions, many patients continue to struggle to afford medicines). 

The cost of many cancer drugs makes for an economic challenge for many countries in Africa, compounded by increased uncertainty about new medicines’ cost-effectiveness. Attempts are being made to minimize some of the risks through the use of international trade agreements [110]. Many of the countries in Africa have fragmented reimbursement systems with multiple actors involved in the purchasing of pharmaceuticals, delivery of health care, and reimbursement mechanisms. Generic pricing policies encouraging local production over imported generics are now seen in Algeria and Egypt. 

In a study comparing the prices of eight expensive cancer therapies, it was found that the UK paid less for the drugs than Argentina, Brazil, Paraguay and Uruguay. Latin America devotes approximately 7.7% of its gross domestic product to health. Except for the widespread inequality and lack of resources for cancer care and control, clinical research in Latin America is highly dependent on funding from pharmaceutical consortia. Sixty-five percent of all clinical trials in Latin America are sponsored by the pharmaceutical industry. In addition, public and private hospitals are not adapted to conduct clinical research, and a long period of time is required for regulatory approval for applications for clinical trials. All of this leads to a reduced interest among pharmaceutical companies in conducting clinical trials in Latin America. There is no HTA system in Colombia, and HTA decisions are based on data from NICE. In Argentina, no centralized/national HTA system is in place [111].

## 5. Discussion

The most evident feature of this panorama is the huge diversity of experience and capacity and outcome on key issues such as NGS, RWE, molecular diagnostics and reimbursement, revealing disparities in access and diagnostics and in knowledge development, screening, and efforts to deepen understanding of cancer. The variability stretches both across and between regions and is accentuated by examination of the performance of individual countries, confirming and complementing many of the findings of the global index created in 2021, which provides a framework for some benchmarking. Regional versions of the BCP or the CM could be envisaged as a pathway to greater collaboration and enhanced common performance—and even perhaps towards a global cancer plan. 

However, what stand out are the widespread inequalities of access to quality care, and the continuing neglect of research into the genomic diversity arising from the ethnic mixes of many countries. This presents a formidable challenge to the exploitation of personalized medicine in general, and in oncology in particular, as without understanding the individual, there is no possibility of precision in adapting diagnostic and therapeutic techniques.

Effective responses need to take on board the many layers of cancer care. Without public education that can dispel myths and misbeliefs about contagion, symptoms, causes, and risks from diagnostic techniques, no amount of technology and medical expertise can improve outcomes. Care options declined are the same as the absence of care options. There is a widespread problem of uneven access to equitable health care and health technology, even when they are provided—a problem posed particularly in remote communities. The wider genetic diversity across the globe has to be recognized so that research includes local populations and development of community-appropriate risk score instruments: European tools have 90% accuracy in European women but only 22% in Asian women, while an Asian Risk Calculator has 71% accuracy in Asian women [112,113]. The economic costs of treatments, techniques and infrastructure remain a major barrier in many countries. Among the priorities to improve outputs are adequate regulatory systems, treatment pathways, specific cancer studies, and training of professionals.

General needed in cancer care improvement are specialized training programs and strengthened education, the creation of local champions, an enhanced role for patient advocacy and empowerment, consistent political support and national health agendas, building up research capacity, and more regional and international and multi-stakeholder collaboration and networking across institutional and disciplinary boundaries, along with education to counter low awareness among the public and patients. Fragmented health care systems, skepticism over the value of PM adoption and limited adoption of EHRs all militate against the most successful evolution. In Table 5, a summary of different key factors in tackling cancer, addressed in different countries, is presented.

A targeted approach would boost awareness and education, promote collaborations and better governance, integrate health tech processes systematically, and aid in providing infrastructure and reimbursement. Solutions proposed during the expert panels focused particularly on high-level and consistent political support, the adoption of national health agendas, a switch in evaluation and regulation to prioritized outcomes over processes, and the development of working groups for regional and international collaboration, built upon a base of multi-stakeholder groups at the country and regional levels.

However, where medical systems focus on more affluent and better served populations, there remains a risk of some distortion of strategy, and where civil society plays little part in decision making processes, this will be slow to change. 

## 6. Conclusions

The conducted review provided very good insights into the current state of cancer care in different regions around the globe. A literature review coupled with expert panels gave us the ability to better identify and evaluate what potentially can be done to bring about a policy framework to tackle cancer, taking into account the local context. Further research is needed per country to ensure that substantive actions are taken to mitigate the impact of cancer and support structural improvements for a more sustainable cancer pathway. 

## Figures and Tables

**Table 1:** Characterization of experts involved in series of panels organized by EAPM.

Feature	Number (%)
Total Number of Experts	48 (100%)
Sex	
Total	48 (100%)
- Female	27 (56%)
- Male	21 (44%)
Specialty	
- Clinician/medical oncologist	16 (33%)
- Regulatory official	11 (23%)
- Patient representative	8 (17%)
- Researcher/geneticist	13 (27%)
Regions	
- Africa	17 (35.5%)
- Asia	17 (35.5%)
- Latam	10 (21%)
- Middle East	4 (8%)

**Table 2:** Cancer situation and related recommendations in different Asian countries.

Country	Cancer Situation	Recommendations
China	➢ country is facing increased cancer rates ➢ many investments in genome science and public health genomics-related programs and services exist ➢ it has national genomics policies to address a variety of genetic issues	➢ there are many clinical resources providing a great foundation to the study of many diseases, including rare diseases ➢ research on biomarkers and big data are developing at a rapid pace within the country
Nepal	➢ health care is underfunded ➢ population suffers from financial risk in the case of using health services for diseases such as cancer	➢ more funding needs to be allocated there ➢ delays in presentation, diagnosis and treatment need to be tackled
United Arab Emirates	➢ the incidence of cancer has increased along with other non-communicable diseases ➢ cancer is the third leading cause of death in the UAE right after cardiovascular disease and trauma ➢ effective referral pathways are lacking and access to diagnostic services is uneven	➢ it is necessary to gather various stakeholders that will cooperate with the goal of successfully creating a policy framework ➢ the demand for pharmacogenomic testing should be higher in clinical practice
Qatar	➢ A National Cancer Research Strategy has transformed cancer care after benefiting from robust governance structures, committed leadership and wide multi-stakeholder involvement ➢ of great interest in Qatar are rare cancers ➢ Qatar Biobank, an initiative created in collaboration with Hamad Medical Corporation and the Ministry of Public Health, has the aim of enabling local scientists to conduct medical research on prevalent health problems in Qatar	➢ integral part of cancer civil society in Qatar is supporting the access to PM and genomic profiling ➢ the aims for the future are to include evidence-based approaches for public engagement, prevention and early detection, especially the use of personalized approaches

**Table 3:** Cancer situation and related recommendations in different African countries.

Country	Cancer Situation	Recommendations
Tunisia	➢ Tunisia runs human genome programs, and the Oncogenetics Unit at the Institut Pasteur in Tunis is conducting research-based genetic diagnosis ➢ a goal to implement PM is set, and a pilot project on lung cancer is already running that covers data generation through to translation in clinical settings	➢ it needs to be foster more awareness of the value of PM, empowering patients, reducing patient payments for testing and drugs, developing infrastructure and HR for tests, sequencing and genotyping facilities along with biobanks ➢ initiation of more local clinical trials
Cameroon	➢ it has a national strategic plan for prevention and cancer control ➢ there is low patient awareness and education, uptake is low, mortality is high (particularly in cervical screening and HPV)	➢ more trained workforce and infrastructure is needed ➢ demand for PM requires further investment from both the public and private sectors and attention from policy makers
Kenya	➢ has set universal health coverage as a priority ➢ moves toward strengthened social health protection schemes are restrained by high cost of health care, poor diagnosis and the emergence of infectious and non-communicable diseases	➢ there is a recognized need for increased stakeholder engagement to boost awareness, skills, ethical/legal frameworks and infrastructure
Nigeria	➢ around 100,000 new cases of cancer occur every year ➢ there is a lack of funding, inadequate prevention, and lack of proper access to basic health care, as well as health-related impoverishment	➢ clinical systems should be integrated into research ➢ education and awareness of patients about novel drugs is needed ➢ immediate availability of novel drugs is needed

**Table 4:** Cancer situation and related recommendations in different LATAM countries.

Country	Cancer Situation	Recommendations
Brazil	➢ the cancer care network in Brazil, in both the public and private sectors, from prevention to palliative care, includes primary care, home care, and specialized outpatient and hospital care ➢ Brazil has been publishing an array of rules to ensure the access of individuals to full care and reduce mortality and cancer-caused impairments ➢ Cancer treatment is performed in specialized care units, including high-complexity oncology centers ➢ Brazil ranks very well in working to reduce smoking rates as a cancer prevention strategy	➢ there should be more data on the mapping, number of patients, and quality of cancer care ➢ taking into account the territorial extent of Brazil, it is even more important and challenging to assure a highly coordinated, multi-layered healthcare system ➢ to increase access to next-generation sequencing in oncology, in Brazil, agencies and payers will be required to collaborate in building data collection infrastructure
Colombia	➢ Colombia had an age-standardized rate (ASR) of 178.8 new cases of cancer per 100,000 people in 2018 ➢ it has a National Cancer Control Plan and aims to: emphasizes cancer prevention; improve early detection; improve quality of cancer care and recovery of cancer patients and survivors; strengthen national information systems; and improve the training and development of practitioners	➢ tackle the challenge related to fragmentation within the health system, resulting in inequitable health outcomes, costs, and quality of services between the public and private systems and among geographic regions
Mexico	➢ cancer is the third leading mortality cause ➢ a small part of the population in Mexico has access to genetic analyses to identify factors associated with the development of some types of cancer, for the early detection of tumors, or to take the option of chemotherapy or prophylactic surgery, which economically would be more affordable ➢ citizens have access to a wide range of special cancer diagnostic preventive measures	➢ find a way to fight the corruption that could be the cause of overpriced medicines, acquiring abroad directly from manufacturers and centralizing their distribution for the entire country, resulting in an enormous shortage of many drugs for cancer and several other medical conditions
Chile	➢ the cancer incidence is substantial, but also many health-related disparities influence distribution of the cancer burden and the quality of cancer care ➢ Chile has witnessed a significant reduction in cancer-related mortality, by 5.2% in women and 5.5% in men ➢ major cancer survivorship programs are lacking ➢ the country has invested in preventive measures such as pap screening tests and an HPV vaccination program	➢ there is a need for well-trained surgical oncologists, in addition to other oncologists ➢ more investment in proper infrastructure and equipment for cancer care is highly needed
Peru	➢ there is a lack of local genomic laboratories ➢ development of a governmental cancer control program and national tumor bank is underway ➢ technology and reagents for NGS are 3 times more expensive than in the US, and most tests (and targeted treatments) are not reimbursed	➢ there is little medical familiarity with biomarkers and genetic tests, highlighting the need for training and public education programs ➢ there is a need for more provision and reimbursement of liquid biopsy (LB) in LC, and more funding of studies of the genomics of cancer in the highly diverse Peruvian population
Venezuela	➢ Overall healthcare spending is around 5% of GDP ➢ Governance of PM in oncology is modest ➢ There is no national cancer institute and plan	➢ greater efforts are needed in adoption and awareness of PM, and availability of education, training and outreach activities is low

**Table 5:** Situation regarding different key factors in tackling cancer addressed in different countries. Countries are listed in alphabetical order regardless of the region.

Countries	Funding of Cancer Treatment/Research	Genomics/Biomarkers	Cancer Incidence and Risk Factors	Cancer Strategic Plans	Primary Prevention Efforts
Angola	It is highly needed to invest in diagnostic facilities, pathology, surgical capacities, chemotherapy, radiotherapy and palliative care resources	Development of genomic surveillance center and lab	Infectious diseases are still of major importance, but mortality rate of oncological diseases is very high	A national plan will be needed to effectively mobilize resources, train staff and reinforce laboratory biosafety measures	More awareness programs and preventive measures need to be put in place
Brazil	National Health System (SUS) subsidizes cancer treatment for approximately 75% of the population; Brazil has the highest public funding for clinical studies	The need to increase access to next-generation sequencing in oncology is recognized, so agencies and payers will be required to collaborate in building data collection infrastructure	Estimations are there were 625,000 new cancer cases in Brazil in 2020	Brazil ranks very well in working to reduce smoking rates as a cancer prevention strategy	The primary care network is the patient’s entryway to the health system in Brazil, playing a decisive role in actions to promote health, prevention and tracking cancer
Cameroon	Funding is limited, and governance and political will to push personalized medicine are lacking	Genetic testing for breast cancer (BC) is out of reach for most patients, and targeted therapies are not available locally; there are unresolved issues on data sharing and on validation and regulatory approval of genetic tests and targeted therapies	Mortality of cancer is high, especially cervical cancer	Cameroon has a national strategic plan for prevention and cancer control, but it makes no reference to personalized medicine, genomics, or biomarkers	There is low patient awareness and education, and uptake of screening is low
Chile	Chile has, after Brazil, the second-highest public funding for clinical studies; Chile has radiotherapy coverage of more than 100%	Tumor sequencing is available in Chile; cancer genomics technologies are not fully implemented; efforts have been made to generate research in gastric, colon and breast cancer	Cancer is the second leading cause of death and accounts for 23.4% of all deaths in the country; most common are prostate, colon, breast, stomach, and lung cancers	Currently, there is no national registry of cancer in Chile, although there are five population-based provincial registries; major cancer survivorship programs are lacking	Access to the primary health-care system is universal and free in Chile. The country has invested in preventive measures such as Pap screening tests and an HPV vaccination program. It also has a program to screen those over 40 with a family history of stomach cancer and a current ulcer and is piloting a screening program for colorectal cancer
China	There are many investments in genome science and public health genomics-related programs and services	Research on biomarkers and big data is developing at a rapid pace within the country; technologies such as NGS and liquid biopsy are widely adopted	China is facing increased cancer rates where the top five commonly diagnosed cancer types in males are lung cancer (14.5%), prostate cancer (13.5%), colorectal cancer (10.9%), stomach cancer (7.2%) and liver cancer (6.3%)	China has national genomics policies to address a variety of genetic issues	Cancer prevention and early action measures are recognized as key projects in Chinese Government; there are many initiatives and guidelines launched
Colombia	There is a need to increase funding to develop innovative cancer technologies, medicines, and treatments accessible to all patients in need	Whole exome sequencing, non-invasive prena taltesting and tumor sequencing are available in Colombia; there is an urgent need to expand the use of NGS in breast, lung, and unknown primary cancers	Colombia had an age-standardized rate (ASR) of 178.8 new cases of cancer per 100,000 people in 2018	Colombia has a National Cancer Control Plan that aims to: emphasize cancer prevention; improve early detection; improve quality of cancer care and recovery of cancer patients and survivors; strengthen national information systems; and improve the training and development of practitioners	There is a universal health care and a government-sponsored 10-year cancer control plan focused on prevention, early detection, and treatment in Colombia
India	Reforms have been put in place that aim to strengthen primary health care and move towards universal health coverage featuring cancer care benefit packages, with a standards-based, interoperable, national digital health information system	Some strengthening of molecular and genomic testing facilities has been triggered by the COVID-19 pandemic	The estimated prevalence of cancer is 2.5 million with an incidence of 0.7 million cases per year. There are some 800,000 new cancer cases in India every year, and tobacco is identified as the most important cause of cancer	India has created a National Cancer Programme envisaging control of tobacco	More efforts in prevention measures should be put in place since the late stage at presentation is very often the main reason for the poor survival from cancer
Kenya	Kenya has set universal health coverage as a priority	Patients have little to no access to genetic testing and counseling services	Cancer is the third leading cause of death after infectious and cardiovascular diseases	Kenya has developed a national cancer control strategy	Public health systems supported by modern technologies and promotion of healthier lifestyles, along with new technologies to enhance disease surveillance, prevention, early diagnosis and treatment are set as priorities
Kingdom of Saudi Arabia	Cancer care is offered free of charge for Saudi patients by a royal decree; more research funding on cancer screening, prevention, and care quality are needed in KSA	Within a period of 5 years, the Saudi Human Genome Program aims to sequence 100,000 samples (normal and disease) from the Saudi population	The incidence of cancer cases and costs of care are high	There are cancer screening programs for breast cancer, colorectal cancer and cervical cancer	The Ministry of Health has been advocating a healthy lifestyle with a healthy diet, physical activity, maintaining ideal body weight, and smoking cessation to decrease noncommunicable diseases, including cancer
Lebanon	Health and third-party payers are providing the financial coverage; cancer drugs are free of charge for uninsured patients, and this country has one of the most developed health care systems in the region	Lebanon is making progress towards implementing precision genetic and genomic research	Cancer rates are increasing and consequently the burden of cancer cost	Many programs put in place raise awareness about cancer screening and prevention through educating and counseling the population, and cancer research has been established by the health ministry	There are cigarette cessation and anti-smoking campaigns for lung cancer as part of preventive measures
Malaysia	Government needs to take a more robust approach to pharmaceutical companies when negotiating prices: at present nearly half of cancer patients experience financial catastrophe within a year of diagnosis	There are challenges regarding NGS testing	Tobacco and infections are reported as the principal causes of cancer deaths, which have a prevalence of close to 1/1000	Malaysia has developed the National Strategic Plan for Cancer Control	There is a need for better awareness and early diagnosis, particularly to enable remote communities to access equitable care with targeted therapies
Mexico	Seguro Popular (SP) was created to provide universal health coverage, including cancer care; 8% of SP’s resources were allocated to Fondo de Protrección contra Gastos Catastróficos (FPGC), of which 28% finances cancer care; about 50–60% of cancer patients in Mexico are fully covered	Mexico has a high number of NGS platforms; a small fraction of the population in Mexico has access to genetic analyses to identify factors associated with the development of some types of cancer, for the early detection of a tumor, or to take the option of chemotherapy or prophylactic surgery	The most common types of cancer among Mexican men are prostate, colorectal, lung, gastric, and testicular at younger ages. Among Mexican women, they are breast, uterine cervix, and colorectal	In 2008, a general law was approved for the control of tobacco and in 2009, in agreement with the General Law Regulation for Control of Tobacco, pictographs and warnings were implemented on the packaging	Genetic counseling and molecular diagnosis are routinely offered by family cancer clinics in a few levels 3 government and specialized private hospitals; citizens have access to a wide range of special cancer diagnostic preventive measures, such as Pap smears offered for women aged 25 to 34 every three years
Nigeria	Nigeria spent only around 0.5% of its 2017 budget on health care; there is a lack of funding	The genomics capacity in Nigeria has for many years been supported by bioinformatics at various institutions across the country	Around 100,000 new cases of cancer occur every year	Nigeria developed the National Cancer Control Plan to reduce the incidence and prevalence of cancer	Prevention measures are inadequate, there is a lack of proper access to basic health care, as well as health-related impoverishment
Nepal	Health care is underfunded, so the population suffers from financial risk in the case of using health services for diseases such as cancer	Genetic research in Nepal heavily relies on resources from international institutes	In 2020, Nepal had an estimated total of 20,508 cancer cases	There is a partially implemented health insurance policy that has several limitations and is not available to everyone and also lacks funding	There are delays in presentation, diagnosis and treatment that need to be tackled
Peru	There is a need for more provision and reimbursement of liquid biopsy (LB) in lung cancer, and more funding of studies of the genomics of cancer in the highly diverse Peruvian population	There is a lack of local genomic laboratories, which means samples requiring comprehensive genomic analysis have to be sent abroad; there is little medical familiarity with biomarkers and genetic tests	In 2020, there were a total of 69,849 cancer cases in Peru	Governmental cancer control program and development of a national tumor bank are underway	There is a need for more public education programs
Philippines	Government is pushed to extend financial and other forms of assistance to impoverished cancer patients and to provide funds for cancer research	There are many genetic tests and services that are available and delivered to the whole country, such as cytogenetics, molecular genetics, biochemical genetics, and newborn screening	Cancer was the second leading cause of death in 2020	Effective screening and prevention strategies exist for many cancers; in February 2019, the National Integrated Cancer Control Act was signed into law	Screening programs and public health education need more efforts
Qatar	Qatar is investing a lot in cancer research; they are bringing in top researchers from all over the world and establishing institutes of research	Cancer molecular genetic boards to integrate PM and genomics into cancer care are now in place.	Projections are that the cancer incidence in Qatar will triple between 2010 and 2030 due to aging and population growth	A National Cancer Research Strategy, Qatar Biobank (QBB) and the Qatar Genome Program (QGP) have been put in place	The aims for the future are to include evidence-based approaches for public engagement, prevention and early detection, especially the use of personalized approaches
South Africa	There are many projects in South Africa that aim to provide better allocation of research funding; the SAMRC is the largest local health research funder in South Africa	There is a lack of population-level genomic data and lack of access to targeted therapies	South Africa has a high burden of noncommunicable diseases (NCDs)	South Africa has established funding of PM, with a genome program and a PM think tank, aiming at a research strategy and product pipeline with an NCD focus on cancer	Cancer prevention guidelines have been put in place; cervical cancer prevention and control policy have been developed
Tunisia	PerMediNA-Precision Medicine in North Africa is linking the Instituts Pasteur in Tunisia, Algeria, Morocco and Paris with EUR 1 million funding from the French government	More sequencing and genotyping facilities along with biobanks are needed	There was a total of 19,446 cancer cases in Tunisia in 2020	Tunisia runs human genome programs, and the Oncogenetics Unit at the Institut Pasteur in Tunis, is conducting research-based genetic diagnosis	Primary prevention strategies remain insufficient as evidenced by the high prevalence of smoking in 2018 (26%)
United Arab Emirates	The top-up funding scheme currently covers breast, colorectal and cervical cancer	There are genomic projects, including the genomic and pharmacogenomic research of the Emirates Genome Program	Cancer is the third leading cause of death in the UAE, right after cardiovascular disease and trauma	National Cancer Control Plan for 2022–2026 has been proposed-and will require accurate data, a reliable cancer registry and periodic monitoring and evaluation	There is a colon cancer prevention program that includes primary preventive strategies and secondary prevention by stool fit test every 2 years or colonoscopy every 10 years
Venezuela	Overall health care spending is around 5% of GDP in Venezuela	Infrastructure in terms of biobank organization is improving	In 2020, there were a total of 58,424 cancer cases in Venezuela	A national cancer plan or strategy needs to be developed	Greater efforts are needed in adoption and awareness of PM; availability of education, training and outreach activities are low

## Data Availability

Not applicable.
